# Valuing and retaining the dental workforce: a mixed-methods exploration of workforce sustainability in the North East of England

**DOI:** 10.1186/s12913-025-12803-9

**Published:** 2025-05-10

**Authors:** Heidi Stelling, Megan Brown, Bryan Burford, Paul Blaylock, Gillian Vance

**Affiliations:** 1 School of Medicine, Newcastle University, Newcastle, UK; 2 School of Dental Sciences, Newcastle University, Newcastle, UK

**Keywords:** Workforce, Retention, Workforce sustainability, Dentistry, Dental, Value, Values

## Abstract

**Background:**

NHS dentistry is experiencing significant recruitment and retention challenges, particularly in rural, coastal, and deprived urban areas. Issues have been exacerbated by the Covid-19 pandemic, leading to unequal distribution of dental professionals across UK geographies. Despite workforce policy initiatives, issues persist. This study explores factors influencing workforce sustainability in the North East of England – an under-served region of the UK.

**Methods:**

Forty-six participants, including 30 dentists, 3 dental care professionals, and 13 managers, contributed to this study. Four focus groups were held at two events in July 2023 – one in the north of the region, and one in the south to enable broad stakeholder engagement and reflect the different geographies within the region. These groups generated qualitative data to elaborate on the factors influencing workforce sustainability and ideas for change. Analysis involved a codebook approach to thematic analysis.

**Results:**

Thematic analysis identified four key factors influencing workforce sustainability: careers, collaboration, costs, and contentment. Career development in a supportive learning environment was essential for professional growth and retention, yet systemic barriers hindered progression. Collaboration, both within dental teams and across regulatory bodies, played a vital role in improving job satisfaction and service delivery, but fragmented communication remained a challenge. Financial pressures, particularly rigid NHS contracts and inadequate remuneration, emerged as significant concerns impacting recruitment and retention. Contentment was shaped by work-life balance, professional recognition, and the ability to provide high-quality care without excessive bureaucracy. These systemic challenges collectively contribute to workforce instability, particularly in the North East.

**Conclusion:**

Findings highlight critical systemic barriers that threaten workforce sustainability in NHS dentistry. Addressing career progression pathways, improving collaboration, reforming contracts, and enhancing professional support systems are essential for sector stability. Without coordinated action from employers and policymakers, NHS dentistry will remain unsustainable, necessitating urgent interventions to support workforce retention and service provision.

**Supplementary Information:**

The online version contains supplementary material available at 10.1186/s12913-025-12803-9.

## Introduction

NHS dentistry is under threat. Across the UK, there are wide-ranging recruitment and retention issues for both dentists, and dental care professionals (including dental nurses, hygienists, therapists, and technicians). There are a variety of challenges which have led to an unequal distribution of dental professionals, with rural and coastal areas being particularly under-served [8]. Workforce shortages have a negative impact on patient care, through reduced access to care and longer wait times for treatment [7]. In deprived areas, where patients’ dental care needs are likely to be higher, the loss of NHS dental care is particularly damaging [8, 19].

Recently, there have been policy changes intended to address the dental workforce crisis. In 2021 Health Education England's Advancing Dental Care (ADC) Review [10] recommended flexible training pathways in dentistry, localised workforce development through apprenticeships, and aligning postgraduate training with areas of the most significant oral health inequalities. In 2023, NHS England unveiled its Long-Term Workforce Plan (LTWP; [14, 15]), which outlines a national strategy for ensuring workforce sustainability, including within dentistry. The plan, which recommends increasing training places for dentists, therapists, and hygienists, and tentatively suggests developing a ‘tie-in’ period where dental professionals would be required to spend a minimum portion of their time delivering NHS care, is a step forwards, but questions remain about its practical application, and feasibility in resolving the crisis.

Recently, retention has emerged as a priority issue. A 2022 survey of 2,204 dentists revealed that 45% had reduced their NHS commitment since the onset of the pandemic [3]. This report also went on to reveal that two thirds of practices have unfilled dental vacancies, with 30% of these in remote, rural, or deprived communities [3]. Further, attrition is affecting the entire dental workforce – 39% of dental nurses have indicated wanting to leave dentistry in the next two years [1].

Our own work [5, 12] has identified challenges to workforce retention and suggested targeted interventions for under-served areas. However, there is ongoing need to understand the work experience of dental professionals, in different roles and settings, in order to design workforce strategies that meet effectively the needs of staff in those communities.

## The North East of England

The North East of England is a large, geographically and socially distinct part of the country, but which contains diverse contexts across the counties of Northumberland and Durham, and the conurbations of Tyne & Wear and Teesside. While it is contiguous with Cumbria, and some areas share similar issues of remote and rural settings, it also has very different urban and coastal settlements, including former mining, fishing and shipbuilding centres, and active commercial ports along its coast.

Indices of deprivation collated by the UK Government [13], further illustrate the distinct context of the North East. Based on income deprivation, of the 12 local authorities comprising the North East in 2019, all but three were in the most deprived quintile, compared to one of the six authorities in Cumbria. The North East contained the most deprived, and the fifth most deprived local authorities in the country. In contrast, two of Cumbria’s local authorities, and none in the North East, were in the least deprived quintile [17]. This highlights how the North East deals with pronounced socio-economic challenges, especially in its urban centres, and particularly in comparison to the more mixed urban–rural characteristics of Cumbria.

Given this inter-regional diversity, the North East is an ideal testbed for studying the dynamics of dental workforce sustainability, and developing transferable recommendations for changes in policy and practice. This paper provides a companion to our earlier work in Cumbria [5], exploring how challenges to recruitment and retention are experienced in a different setting. As in the Cumbria paper, our interest was in all staff working in dental primary care. This included all dental professions required to be registered with the General Dental Council (GDC; dentists, and dental care professionals (DCPs) including dental nurses, dental hygienists, dental therapists, orthodontic therapists, dental technicians, and clinical dental technicians), and non-clinical staff in support and managerial roles, such as practice managers and receptionists. These non-clinical roles are essential for the effective running of dental practices, and so the effective delivery of care.

The work on which this paper is based was commissioned by Health Education England, which merged with NHS England in 2023. It aimed to investigate the current state of the multi-professional dental workforce in both general and community dental services in the North East. This paper addresses two research questions:

1. What factors influence the career decisions of dental professionals currently working in the North East?
2. What strategies may improve recruitment and retention of dental professionals in the North East?

## Methods

This study incorporated both an individual and practice survey (Supplementary file 1 and 2) and focus group component, with the complete results available in Burford et al. [5]. The following section presents only the qualitative data obtained from the focus groups.

### Data collection

Four focus groups were held at two events in the North East in July 2023 – one in the north of the region, and one in the south to enable broad stakeholder engagement and reflect the different geographies within the region. The outline for the workshops is provided in Supplementary file 3.

These groups generated qualitative data to elaborate on the factors influencing workforce sustainability and ideas for change. The ‘driver diagram’ (Supplementary file 4), commonly employed in quality improvement practices [16] served as a focal point for workshop discussions. The research team provided facilitators and participants with a copy of a blank driver diagram as a visual cue to focus attention on the ‘drivers’, or causes, that might facilitate or hinder the desired outcome of improving dental care in the North East. In addition, the driver diagram directs participants to consider strategies, or the solutions, required to have a positive impact on identified drivers.

All members of the research team acted as facilitators or co-facilitators, with co-facilitators taking detailed notes regarding discussions and analytical thoughts. Discussions ranged between 55 and 67 min.

### Analysis

Audio recordings were manually transcribed verbatim to facilitate analysis. Braun and Clarke’s [2] codebook approach to qualitative analysis was followed, a middle-ground between highly structured analyses, and fully exploratory methods. Our codebook approach allowed us to organise data using a systematic structure but also remain attuned and sensitive to new insights grounded in our participants’ experiences.

Our codebook was created from codes utilised within our previous 2022 Cumbria report [5], given the fact this study builds on these previous findings at a regional level.. Researchers (HS, MB, BB, GV) collaboratively applied the codebook to the data, allowing for its refinement. Descriptions of each code were included to ensure different researchers could code transcripts in similar ways. These codes are listed in full, with their corresponding descriptions, in Supplementary file 5. To ensure coherency and transferability of the codebook, two transcripts were double coded, and outputs compared. The codebook was used to organise all data, connecting it to create a thematic narrative, and extracting relevant illustrative quotes. Results were discussed, agreed, and presentation finalised by all authors.

### Reflexivity

Our research team consists of experienced qualitative and mixed-methods researchers and dental workforce experts from Newcastle University. Our team includes clinical academics, and academics with backgrounds in medicine, medical education, dentistry, and workforce policy. We are a mixture of genders and ages. We acknowledge that our professional experiences and prior research on workforce sustainability may have influenced our interpretation of the data. To this end, we held regular discussions to support reflexive results interpretation throughout analysis.

### Quality

To enhance the transparency and completeness of our study reporting, the Consolidated Criteria for Reporting Qualitative Research (COREQ) checklist [20] has been used. The COREQ checklist provides a structured framework for supporting rigor in qualitative research and can be reviewed in full in Supplementary file 6.

## Results

Thirty dentists, 3 dental care professionals, and 13 managers participated in our four focus groups (46 participants in total). Four themes were generated by the analysis to capture key findings:

1. Careers: Career development, nurtured in a safe, supported, and learning-focussed space, enables professional advancement.
2. Collaboration: Working collaboratively improves oral health outcomes, which in turn supports a sustainable dental workforce.
3. Costs: Fair pay and contracts, guided by policies, play a role in retaining dental professionals.
4. Contentment: Dental professionals’ satisfaction with their jobs leads to a more sustainable workforce.

Participant quotes are labelled with focus group number (e.g., FG1), and participant number within the focus group (e.g., P5).

### Careers

Across professional groups, staff were motivated to develop and learn. This learning was even more critical against a backdrop of increasingly complex patient care post-Covid-19. Cultures supportive of learning were positive driving factors towards workforce sustainability.

For example, practitioners described wanting to diversify their skills and achieve more variety in their working week.

*FG1 P3: “…retention comes with variety and if you haven't got variety, you're struggling. [It] goes back to changes in NHS practice… you go in and you're just doing amalgams or extractions, people don't want that anymore.”*

Many felt strongly about the need to host undergraduate students in general dental practices, noting how this would benefit the career insights of the student and could stimulate interest in teaching amongst the staff.

*FG4 P2: “Nurses would enjoy the variety of working with a student.”*

Similarly, being an Educational Supervisor for the Foundation Dentist [FD] programme was viewed positively. However, the national recruitment process was a source of concern. Being ‘allocated’ a Foundation Dentist removed the autonomy of practices around recruitment. The autonomy to establish a team based on ‘fit’ and trust provided a solid foundation for a positive working environment. The challenges of national recruitment and poor staff allocation were highlighted, particularly in relation to their disproportionate impact on dental practices as small businesses, where a single unsuitable hire could jeopardise the practice's stability and success.

*FG4 P6: “[National recruitment] could break your practice... If I'm a hospital consultant and my trainee is not very good when they've left, I'm still a hospital consultant.”*

Participants discussed additional barriers to the career development of all professional groups. Many of these related to practical issues, such as costs (of training, and from loss of income in attending an event), and a lack of physical space and time to facilitate best use of skill-mix.

*FG1 P5: [They (DCPs) have] additional skills that can be used utilised. But I haven't got a room for you to see patients to do oral health education. If I did, I would pay you to do it. I physically don't have space.”*

Participants expressed desires to create learning-focused cultures, where open dialogue about mistakes, challenges, and improvement was encouraged.

*FG4 P6: “We sat and we problem solved. And we talked about your difficult cases. Which is great to do, and that's fewer complaints and better dentistry and everybody wins on that.. So it's a really positive thing to do.”*

### Collaboration

Collaborations were central to enhancing the experience of dental professionals, shaping clinical practice, and optimising care. This theme explores the nature of collaboration, and its relationship with dental workforce sustainability.

Participants identified the importance of creating strong professional relationships with other team members. This was particularly critical for those new to NHS dentistry or considering leaving it. Establishing these connections was seen as essential in providing the necessary support, guidance, and career development opportunities to help retain staff. Measures that facilitated mentoring, open discussions about challenges, and targeted professional development were viewed as key to sustaining engagement and confidence within the workforce.

*FG4 P6: “So what do I do if I've got a group of associates who are really struggling to hang on to NHS dentistry, I take them out to do that and I say, where are your problems? How can I assist? How can I support? How can I give you that career development support that you need?”*

Some participants described the loneliness of working in remote or small practices, and expressed a desire for regional networking events that would encourage professional dialogue and social connection.

*FG4 P5: “Attracting people away from the cities where their comfort zone is, where their social network might be and where their peer network is. And if we had a more formal structure of professional networking around [we] might be more attractive to be able to help us recruit in those areas”*

There was a suggestion that a more collaborative relationship with commissioners and regulators built on shared understanding and mutual respect would enhance workforce sustainability. For example, improved lines of communication between agencies (for example, NHS-England and the Care Quality Commission) could dramatically reduce the administrative burden created through duplication of documentation in NHS dentistry.

*FG2 P4: “It just seems to be a lot of duplicate work and nobody's sharing the information with each other. So then we have to submit it again…You don't have to do that if you're a private practice.”*

Collaboration also extended to the public and ways of changing negative perceptions of dentistry and oral health. Many participants emphasised their willingness to create meaningful, and important connections within local communities and cited an example of how positive an outreach education activity had been for a staff member. However, these motivations were hindered by systemic barriers (e.g., financial constraints).

*FG2 P7: “I went to a school the other day and it was the best day of my life…[But] there's no funding for it... It's all based around private dentistry, earning the money, earning the money. There's no community. There's no education.”*

### Costs

Funding issues – relating to financial pressures, commissioning, and contractual constraints – were a large focus of discussions. These factors were central to the operational viability of dental practices and the retention of practitioners within the NHS.

The NHS General Dental Services (GDS) contract was negatively perceived. One participant described a colleague earning more money outside of dentistry, communicating a sense of unfairness about the current system of renumeration.

*FG2 P4: “I've got a dentist who worked full time and has reduced her NHS commitment to not go and work in a private practice. She's actually opened a pizza restaurant and is earning more money through the pizza restaurant than she is in, in dentistry.”*

Commissioning was felt to be too restrictive, failing to value health promotion. The current contractual framework, with a pressure to deliver mandatory services as Units of Dental Activity (UDAs), discouraged use of skill-mix or any professional activities outside of direct patient care. Participants described feeling like a *“hamster on the wheel” [FG1 P5]* constantly chasing ‘UDAs’.

Additionally, contractual arrangements meant that practices sometimes felt restricted in hiring Foundation Dentists: they could not justify the space needed for a Foundation Dentist when their UDAs did not count towards practice targets. Practices accepted this was short-sighted for workforce sustainability, but the immediate pressures for the practice to survive outweighed longer-term concerns.

*FG2 P4: ”In my practice, I can't afford to have a surgery given to an FD because of their UDAs, we have such a high target their UDAs don't count towards the targets. I couldn't afford to have an FD in there.”*

There was broad agreement that additional funding is required to sustain NHS dentistry. While practitioners may remain committed to NHS *clinical* care, practice managers saw NHS *business* as being financially non-viable. There was a clear tension between the provision of NHS care and the functioning of a practice as a viable business.

*FG2 P4: “[I’m] pushing to say the practice cannot continue as an NHS practice. Financially, it can't do it. The accountants are telling us, the bank is telling us, I'm telling them, they're [practitioners] are the ones who are telling me saying, no, we want to stay NHS… I keep telling them that we're not charity workers.”*

### Contentment

The multi-dimensional factors that contribute to, or detract from, contentment, and the implication of these factors for workforce sustainability are explored in this theme. This section also explores the close relationship between contentment and the decision to transition to private practice.

Central to this theme was the recognition that living and working in the North East offers a high ‘quality of life’. Many felt this could be better promoted.

*FG4 P1: “The North East is a great place to live and work and we should acknowledge that… well supplied with schooling, housing, interconnected transport links…If we’re looking to enhance the workforce, we should be selling that.”*

There was pride in working for the NHS, especially relating to the provision of continuity for patients and communities. This pride was central to contentment and fuelled practitioner resilience.

*FG2 P4: “They've seen those patients grow up from being a child to an adult who's bringing their kids to the practice now…if it was money orientated, they would have jumped ship a long time ago.”*

However, several barriers to job satisfaction or contentment were identified, including challenges in maintaining work-life balance, lack of career opportunities in the North East for practitioners’ family members, and not feeling valued in the same way as other NHS staff.

*FG2 P8: “I think people were quite put down during Covid because dental nurses were not considered part of NHS, because there were all these NHS perks which was not available for dental nurses.”*

In relation to private practice, many seemed defensive regarding their engagement, and eager to express that the primary incentive for transitioning to private practice was not always financial. Rather, it was frequently the desire to sidestep the bureaucratic burden of the NHS and provide higher quality care for patients.

*FG4 P5: “It's stick after, stick after stick after stick. And as a practice owner you go, why am I putting myself through all of this regulation when with one simple manoeuvre I go, I'm not playing this game anymore?”*

## Discussion

The North East presents unique challenges for the dental workforce, with our findings identifying multiple factors influencing recruitment, retention, and workforce distribution. This discussion reflects on these findings, considering key implications for policymakers and employers. These new findings allow for the development of recommendations that build on our earlier dental workforce work in Cumbria, which identified measures to improve recruitment and enhance individuals’ sense of purpose at work. This paper extends those recommendations by offering a more in-depth analysis of retention, and focusing on system-level solutions, which are necessary to sustain NHS dentistry in the region.

### Feeling valued as a professional

A key factor influencing sustainability was the extent to which dental professionals felt valued, both financially, and professionally. While job satisfaction and career development opportunities are important across all healthcare roles, these findings suggest that in the North East, where workforce shortages are acute, feelings of being undervalued exacerbate attrition. Younger dentists and DCPs were often demotivated because of limited opportunities for progression, and the absence of formal support programmes. This echoes our findings in Cumbria [5], where dental professionals felt isolated, and wider literature showing high attrition rates among young dental nurses, for whom progression opportunities are especially limited [9]. Importantly, these frustrations contribute to challenges of workforce distribution, as professionals opt to leave NHS practice for private roles, or relocate to regions perceived as offering better career prospects.

A significant finding was the perception that NHS dental professionals, particularly dental nurses, were not fully integrated into the wider NHS workforce. Unlike their counterparts in other NHS settings, many dental nurses lack access to NHS pensions and other benefits. The impact of this as a barrier may be particularly pronounced in the North East (indeed, this was not found in the earlier work in Cumbria), given high levels of income deprivation in the North East which may make NHS employment benefits critical. Some nurses therefore seek employment in hospital-based roles, or outside the NHS altogether. Addressing these disparities requires systemic change, including policy interventions to extend NHS employment benefits to all dental professionals and ensure parity with other NHS roles.

### Collaboration and workforce sustainability

Our findings reinforce that a strong, collaborative team culture is vital for retaining dental professionals. Participants described how mentorship and professional networking could mitigate feelings of isolation, particularly for those working in smaller, remote practices. A lack of regional networking opportunities was cited as a reason for leaving NHS roles in the North East, with some professionals relocating to more urban areas where stronger professional support systems exist. While previous research [9] has identified collaboration as a positive workplace factor, our study highlights its direct impact on regional workforce distribution – practitioners are more likely to remain in NHS roles if they feel professionally connected and supported.

Additionally, these findings indicate that systemic collaboration – between dental professionals, commissioners, and regulators – could alleviate some of the administrative burdens driving workforce attrition. Participants expressed frustration with duplicated regulatory processes, which increase workload and reduce job satisfaction.

While specific administrative challenges have been documented for international dentists navigating credentialing and licensing [6], and for international medical educators where heavy administrative burden contribute to work-life imbalance and burnout, negatively affecting retention [18], the impact of varying administrative burdens on retention within NHS dentistry has not yet been explored. Consideration of processes and policy measures that streamline administration, reduce duplicative regulatory requirements, and improve communication could significantly improve efficiency and enhance job satisfaction.

### Financial pressures and workforce distribution

In relation to financial considerations, our participants reported a lack of flexible commissioning and rigid contract terms that contributed to feelings of being undervalued. The NHS General Dental Services (GDS) contract was frequently criticised by participants for discouraging skill-mix utilisation and creating financial pressures on practices. While dissatisfaction with NHS contracts is a widespread issue, these findings suggest a direct link between contractual rigidity and workforce distribution. In the North East, where NHS dental contracts are often seen as less financially attractive due to socioeconomic factors, practices struggle to retain staff. For example, some participants reported their practices could no longer justify hiring Foundation Dentists due to contract constraints, despite recognising that failing to do so would have long-term negative implications for workforce sustainability. These financial pressures contribute to uneven workforce distribution, as practices in deprived or rural areas struggle to compete with private sector opportunities.

A shift towards flexible commissioning models [14] that recognise the diverse range of services provided by dental practices, and support skill-mix, could significantly improve care as illustrated in Yorkshire and the Humber, where flexible commissioning led to an increase in appointments and range of services offered [11].

### Implications

Our findings identify several important, practical recommendations for policymakers and employers, as summarised in Table 1.

**Table 1:** Recommendations for policy and practice, based on this study’s findings

Recommendation	Detail	Theme Mapping
Structured mentorship	Consider introduction of accessible opportunities for mentorship, especially for early career professionals. Formal support, including support for career progression, may help overcome feelings of demotivation amongst younger dental professionals	Collaboration, careers
Policy changes to NHS employment benefits	Review access to employment benefits for dental staff to ensure parity across all NHS professionals and improve retention in underserved areas	Contentment
Contract reforms	Continue to lead review and reform of the NHS GDS contract to reflect the diverse workload, skills, and experience of dental professionals more accurately. Consider flexible models of commissioning	Cost, careers
Administrative streamlining	Review and reduce administrative burdens in NHS dental care to improve efficiency and service delivery	Collaboration
Professional networking opportunities	Take steps to create a strong, collaborative team culture not just within individual practices, but regionally by establishing professional networks that all dental professionals are supported to engage with. Create both professional and social opportunities for support and connection-building	Careers, collaboration

### Limitations

With focus groups generally there is the risk that those with strong opinions may be more likely to participate, leading to potential self-selection bias. In this study, where data collection was conducted at specific workforce events, dental professionals with knowledge of and interest in workforce issues, may have been overrepresented. Their views may not fully reflect those of the broader workforce. Additionally, the focus groups lacked balance with only three DCPs, meaning that the interests and experiences of DCPs may be underrepresented, although dentists and practice managers did present DCP viewpoints.

The analysis did not attribute quotes to specific occupations, making it difficult to link statements to professional perspectives. However, the strength of this study lies in the richness of our data, which presents valuable insights into the experiences of dental professionals in the North East. The findings are considered transferable to other regions with diverse geographies and demographics, given the participants'diverse cross-professional backgrounds and observations consistent with broader literature. Future research should focus on exploring national applicability.

## Conclusions

These findings underscore systemic barriers that jeopardize workforce sustainability in NHS dentistry. Strengthening career progression pathways, improving collaboration, and addressing financial constraints are critical to retaining a skilled and motivated workforce. Meaningful reform requires coordinated action from policymakers, employers, and regulatory bodies to create an environment where dental professionals can thrive. Without urgent intervention, NHS dentistry risks continued workforce decline, further limiting access to essential care for diverse communities.

## Supplementary Information

Supplementary Material 1.

Supplementary Material 2.

Supplementary Material 3.

Supplementary Material 4.

Supplementary Material 5.

Supplementary Material 6.

## Data Availability

The raw datasets generated and analysed during the current study are not publicly available due to the fact that ethical approval and consent was not obtained for data sharing publicly but are available from the corresponding author on reasonable request.

## Abbreviations

ADC: Advancing Dental Care
CPD: Continuing Professional Development
DCP: Dental Care Professionals
FD: Foundation Dentist
GDS: General Dental Services
LTWP: Long Term Workforce Plan
NHS: National Health Service
PG: Postgraduate
UDA: Units of Dental Activity
UG: Undergraduate
