# Supplementary material for: Valuing and retaining the dental workforce: a mixed-methods exploration of workforce sustainability in the North East of England
Source: BMC Health Serv Res. 2025 May 10;25:672. doi: 10.1186/s12913-025-12803-9 (PMC12065166; doi:10.1186/s12913-025-12803-9)
Supplement: Supplementary file 1 — Supplementary Material 1. [file 12913_2025_12803_MOESM1_ESM.pdf]

North East Dental Workforce Survey 2023 - individual survey

Page 1

To help understand some of the issues behind the current crisis in the dentistry workforce, Newcastle University School of Medicine, supported by NHS England Workforce Training and Education North East, is conducting research into the dental workforce across North East England.

As part of this, we are asking dentists, dental care professionals and others working in dentistry about their career plans and the influences on those plans. This will help us understand if more could be done to encourage people to work in the area.

Your responses will be completely anonymous, and will be stored securely at Newcastle University. Individual responses will not be shared with anyone outside the university.

If you would like to be entered into a draw for a £100 gift card, then please enter your email address at the bottom of this page - this will be removed from your other responses and not used to identify you.

If you have any questions about the survey, please contact bryan.burford@newcastle.ac.uk.

Your responses will not be recorded until you click 'Finish' at the bottom of the page.

1. What is your job

- Dentist
- GDC registered dental nurse
- Dental therapist
- Dental hygienist
- Dental nurse in training

- ○ Orthodontic therapist
- ○ Practice manager
- ○ Receptionist
- ○ Other role (eg dental technician)

1.a. If you selected Other, please specify:

[ ]

2. What is the local authority of the practice where you work?

[ ]

2.a. Please state the local authority

[ ]

2.b. How long have you worked in this location?

[ ]

3. Which sector do you work in? (tick all that apply)

- ☐ General dental practice (private only)
- ☐ General dental practice (NHS or NHS and private)
- ☐ Community dental services
- ☐ Secondary care
- ☐ Other

3.a. If you selected other, please give some detail

4. **What attracted you to work where you do? (Please select all that apply)**

- ☐ Born/grew up in the area
- ☐ To be near to family
- ☐ To be near to friends
- ☐ Attracted to a rural setting
- ☐ Having access to cities
- ☐ Having access to the coast
- ☐ Particular interest in a job/training opportunity
- ☐ Another reason(s)

4.a. If you selected 'Another reason(s)', please give details:

5. Please indicate whether you are considering any of the following. (By 'considering' we mean you have given serious thought to, but need not have made any plans).

	Not applicable	I am not considering this	In the next 6 months	In the next year	In the next 5 years

Reducing your working hours in your current job	☐	☐	☐	☐	☐
Changing the mix of NHS/private work in your job	☐	☐	☐	☐	☐
Leaving your current job for another job in dentistry in the north east	☐	☐	☐	☐	☐
Leaving your current job for another job in dentistry in another region	☐	☐	☐	☐	☐
Leaving your current job for further training in dentistry	☐	☐	☐	☐	☐
Leaving dentistry (other than through retirement)	☐	☐	☐	☐	☐
Retiring	☐	☐	☐	☐	☐

6. Thinking about your responses to question 4, what influences you to consider changing, or staying in your current job? This may include personal (eg financial, housing) as well as professional reasons.

The demographic questions below will help us have a better understanding of the composition of the workforce in different staff groups. This information will not be used to identify you personally, and will be held securely by Newcastle University.

7. Are you

- ○ Male

- Female
- Other
- Prefer not to say

8. What age are you

- Under 20
- 20-29
- 30-45
- 46-55
- Over 55

9. What is your ethnic group? (This question is based on the UK census from 2021)

- White (Includes British, Northern Irish, Irish, Gypsy, Irish Traveller, Roma or any other White background)
- Mixed or Multiple ethnic groups (Includes White and Black Caribbean, White and Black African, White and Asian or any other Mixed or Multiple background)
- Asian or Asian British (Includes Indian, Pakistani, Bangladeshi, Chinese or any other Asian background)
- Black,Black British, Caribbean or African (Includes Black British, Caribbean, African or any other Black background)
- Other ethnic group (Includes Arab or any other ethnic group)

9.a. Which best describes your White ethnic group or background?

- British
- Northern Irish
- Irish

- Gypsy
- Irish Traveller
- Roma
- Any other White background

9.b. Which best describes your Mixed or Multiple ethnic group or background?

- White and Black Caribbean
- White and Black African
- White and Asian
- Any other Mixed or Multiple background

9.c. Which best describes your Asian or Asian British ethnic group or background?

- Indian
- Pakistani
- Bangladeshi
- Chinese
- Any other Asian background

9.d. Which best describes your Black, Black British, Caribbean or African ethnic group or background?

- Black
- Black British
- Caribbean
- African
- Any other Black background

9.e. Which best describes your other ethnic group or background

- Arab
- Any other ethnic group

9.f. Please provide more information about your ethnic group if you would like to.

10. If you would like to be entered into a draw for a £100 gift card, please give your email address here. This will be removed from your questionnaire data, and not linked to your responses.

Page 2: Final page

Thank you for completing the survey.

If you have any questions about it, please contact bryan.burford@newcastle.ac.uk.

Key for selection options

2 - What is the local authority of the practice where you work?

- County Durham
- Darlington
- Gateshead
- Hartlepool
- Middlesbrough
- Newcastle
- North Tyneside
- Northumberland
- Redcar and Cleveland
- South Tyneside
- Stockton on Tees
- Sunderland
- Other
