# Supplementary material for: Valuing and retaining the dental workforce: a mixed-methods exploration of workforce sustainability in the North East of England
Source: BMC Health Serv Res. 2025 May 10;25:672. doi: 10.1186/s12913-025-12803-9 (PMC12065166; doi:10.1186/s12913-025-12803-9)
Supplement: Supplementary file 2 — Supplementary Material 2. [file 12913_2025_12803_MOESM2_ESM.pdf]

North East dental workforce survey 2023 - practice survey

To help understand some of the issues behind the current crisis in the dentistry workforce, Newcastle University School of Medicine, supported by NHS England Workforce Training and Education North East, is conducting research into the dental workforce across **North East England** following a similar survey in Cumbria in 2022**.**

This survey asks about the composition of the workforce in **primary care dentistry across the North East of England -** in **private and NHS sectors.** Results will support the planning of education and training for the dental workforce needs of the future.

This questionnaire should be completed with details of staff for a **single practice location** (if part of a partnership or group, each location should return a separate questionnaire).

We expect it to take up to 20 minutes to complete, but suggest you make sure you have information on the practice staff to hand. To preview the questions and check you have the information available, you can view a pdf of the survey by following this link.

If you don't have access to this information, please forward the survey link (https://newcastle.onlinesurveys.ac.uk/dental-workforce-practice-survey) to someone who will.

If you have any questions about the survey, please contact Bryan Burford at Newcastle University (bryan.burford@newcastle.ac.uk).

Practices which complete the survey will be entered into a prize draw for £400.

Your responses will not be recorded until you click 'Finish' on the final page. If you wish to return to complete the survey later, use the 'Finish later' button on each page.

Please select the local authority in which your practice is located, and then select your practice from the pop-up list. If the practice has '(completed)' next to it, someone from your practice has already responded and you do not need to respond again (unless you wish to correct or update information).

This information comes from the Care Quality Commission register, your practice may be known by a different name. If that is the case, please select the option which represents your practice, but give its usual name in the text box below.

Some practices may be in a different local authority to their postal address.

In which local authority is your practice?

- County Durham
- Darlington
- Gateshead
- Hartlepool
- Middlesbrough
- Newcastle
- North Tyneside
- Northumberland
- Redcar and Cleveland
- South Tyneside
- Stockton on Tees
- Sunderland

Please select your practice

Please select your practice

Please select your practice

Please select your practice

Please select your practice

Please select your practice

Please select your practice

Please select your practice

Please select your practice

Please select your practice

Please select your practice

Please select your practice

If your practice is not listed in the pop-up list above, please give the name and full postcode here.

Please enter the name and contact details of the person completing this survey. We will only use this if we require clarification of any responses.

NHS services and ownership

Does this practice currently treat patients...

- ◌ Exclusively privately
- ◌ Exclusively NHS
- ◌ A mixture of private and NHS

Please give the approximate percentage of NHS care

[ ]

Does the practice have plans to change its mix of NHS/Private work in the next 12-18 months?

- ◌ Increase % of NHS care
- ◌ Decrease % of NHS care
- ◌ Stop providing NHS care
- ◌ Stop providing private care

Does the practice have plans to change its mix of NHS/Private work in the next 12-18 months?

- ☐ Start providing NHS in addition to private care
- ☐ Stop providing private care

Does the practice have plans to change its mix of NHS/Private work in the next 12-18 months?

- ☐ Start providing private in addition to NHS care
- ☐ Stop providing NHS care

Is this practice independent or a member of a corporate/group provider?

- ◌ Independent (single owner)
- ◌ Independent (partnership)
- ◌ Corporate/group

Which corporate/group is the practice part of?

[ ]

Numbers of dentists

Please complete the table below for all dentists working in your practice.

How many dentists (including Foundation Dentists) work in your practice. ✱ *Required*

- ○ 1-6
- ○ 7-12
- ○ 13-18

Details of individual dentists

	Sex	Age group					Normal weekly hours	Approx NHS workload (%)	Is this a GDC-registered specialist?	Is this a Foundation Dentist?
		Under 30	30-45	46-55	Over 55	Not known				
Dentist 1										
Dentist 2										
Dentist 3										
Dentist 4										
Dentist 5										
Dentist 6										

Details of individual dentists

	Sex	Age group: Under 30	Age group: 30-45	Age group: 46-55	Age group: Over 55	Age group: Not known	Normal weekly hours	Approx NHS workload (%)	Is this a GDC-registered specialist?	Is this a Foundation Dentist?
Dentist 1										
Dentist 2										
Dentist 3										
Dentist 4										
Dentist 5										
Dentist 6										
Dentist 7										
Dentist 8										
Dentist 9										
Dentist 10										
Dentist 11										
Dentist 12										

Details of individual dentists

	Sex	Age group					Normal weekly hours	Approx NHS workload (%)	Is this a GDC-registered specialist?	Is this a Foundation Dentist?
		Under 30	30-45	46-55	Over 55	Not known				
Dentist 1										
Dentist 2										
Dentist 3										
Dentist 4										
Dentist 5										
Dentist 6										
Dentist 7										
Dentist 8										
Dentist 9										
Dentist 10										
Dentist 11										
Dentist 12										
Dentist 13										
Dentist 14										
Dentist 15										
Dentist 16										
Dentist 17										
Dentist 18										

Numbers of other staff groups

	How many staff do you employ in this group?	What is the whole time equivalent number of staff in this group (this may be less than the total number of staff if people work part time)?	Number who are Male	Number who are Female	Number aged under 30	Number aged 30-45
Dental therapists						
Dental Hygienists						
GDC registered dental nurses*						
Dental nurses in training						
Orthodontic therapists						
Practice managers						
Receptionists						
Other roles (eg dental technicians)						

*** Excluding qualified dental nurses whose main role is in another job - eg practice manager or receptionist.**

Please provide details of any roles included under 'Other' above

For each of the following groups please indicate any vacancies or retention issues.

	Current vacancies	Ongoing problems with retention or high turnover
Dentists	☐	☐
Dental therapists	☐	☐
Dental Hygienists	☐	☐
GDC-registered dental nurses	☐	☐
Dental nurses in training	☐	☐
Orthodontic therapists	☐	☐
Practice managers	☐	☐
Receptionists	☐	☐
Other roles (eg dental technicians)	☐	☐

Please tell us more about any recruitment or retention issues

Place of qualification

Please indicate the numbers of each staff group who obtained their primary dental qualification in each area.

	North East England	North West England	Elsewhere in the UK	Elsewhere in the European Economic Area	Elsewhere in the world	Not known
Dentists						
Dental therapists						
Dental Hygienists						
GDC registered dental nurses						
Dental nurses in training						
Orthodontic therapists						
Other roles (eg dental technicians)						

Details of dental nurse training

How many dental nurses in this practice location have or are in training for the following primary dental qualifications?

	Have qualification	In training
National Examining Board for Dental Nurses (NEBDN) National Diploma in Dental Nursing		
City and Guilds NVQ Level 3 diploma in Dental Nursing		
Other primary dental nursing qualification		

How many dental nurses in this practice location entered the GDC register through 'grand-parenting' when registration was first introduced.

How many dental nurses in this practice location have or are in training for the following qualifications (NEBDN or other)?

	Has NEBDN qualification	Has other qualification	In training
Dental Implant Nursing			
Dental Radiography			
Dental Sedation Nursing			
Oral Health Education			
Orthodontic Dental Nursing			
Special Care Dental Nursing			

Services provided in the practice

Which of the of the following activities are undertaken by the following staff groups at any time in this practice location? Please consult with the Principal Dentist or Practice owner to complete this table if necessary.

	Dental Therapist	Dental Hygienist	Dental Nurse (including joint receptionist or practice manager roles)	Orthodontic Therapist	Other
Prescribe radiographs	☐	☐	☐	☐	☐
Take radiographs	☐	☐	☐	☐	☐
Apply fluoride varnish to teeth	☐	☐	☐	☐	☐
Deliver Oral Health Education	☐	☐	☐	☐	☐
Take impressions	☐	☐	☐	☐	☐
Give smoking cessation advice	☐	☐	☐	☐	☐
Other	☐	☐	☐	☐	☐

Please provide details of any 'Other' responses above.

Approximately what percentage of dental therapists' time is spent performing hygienist activities or competencies?

Which of the following services are provided at this practice?

	Provided by a non-specialist dentist	Provided by a GDC-registered specialist dentist
Implants	☐	☐
IV sedation	☐	☐
Orthodontic services	☐	☐
Inhalation sedation	☐	☐
Minor oral surgery (excluding simple exodontia)	☐	☐
Domiciliary care	☐	☐
Other (please specify	☐	☐

If you have selected other, please provide details here

What postgraduate qualifications (eg MSc, MFDS, MJDF) are held by dentists working in this practice (if known)?

Other information

Please tell us how professional development or learning needs amongst your staff could be better supported.

If relevant, what might encourage you to become a Dental Foundation Training practice? Please provide as much detail as you think is relevant.

Finally, please add any other information that may help us interpret the shape and needs of the dental workforce. For example, what growth or future employment plans may you have in the practice?

Final page

Thank you for completing the questionnaire.

If you have any questions about the survey, please contact Bryan Burford at Newcastle University (bryan.burford@newcastle.ac.uk)

We are also running a separate survey about individual staff member's career plans. This can be found at this link: https://newcastle.onlinesurveys.ac.uk/dental-workforce-individual-survey

Please feel free to forward this to any colleagues working in dentistry in the North East.

Key for selection options

1.a - Please select your practice

(completed) Bupa Dental Care Darlington, First Floor, 20 Woodland Road, Darlington
Cleveland Terrace Dental Practice, 8 Cleveland Terrace, Darlington
Darlington Dental Clinic, 18 Southend Avenue, Darlington
Duke Street Dental Practice, 44 Duke Street, Darlington
Harrowgate Hill Dental Practice, North Road, Darlington
(completed) Lance Robsons Dental Practice
MSG Dental Care Limited, Middleton Lane, Middleton St. George
Neasham Road Dental Practice, Neasham Road, Darlington
Phillips & Co Cosmetic Dentistry, 15 Woodland Road, Darlington
Queensway Oral Surgery-Darlington, 293 Yarm Road, Darlington
Queensway Orthodontics West Limited, 2b Duke Street, Darlington
S Leigh Dental Surgery, Stockton Road, Darlington
Skinnergate Dental Practice, 76 Skinnergate, Darlington
Smile@Westpark Dental Studio, West Park
Springs Dental Studio, 146 Yarm Road, Darlington
Toothwise - Hurworth Dental Practice, Church Row, Hurworth
Toothwise - Southend Avenue Dental Practice, 12 Southend Avenue, Darlington
Victoria Dental Practice, Victoria Road, Darlington

1.b - Please select your practice

Alan Lam Family Dental Practice, Weardale Terrace, Chester Le Street
All Smile Dental, South Burns, Chester Le Street
(completed) Alpha Dental Studio, Chester Le Street
Ashfield Dental Care, Ashfield Terrace, Chester Le Street
Auckland Dental Practice, 44 Cockton Hill Road, Bishop Auckland
Aycllife Dental Practice, Faulkner Road, Newton Aycliffe
Belmont Dental Practice, Belmont, Durham
Bennisons Dental Practice, Lowland Road, Brandon
Bowburn Dental Health Centre, Wellsprings Business Centre, Bowburn
Bupa Dental Care Bishop Auckland, 69 Cockton Hill Road, Bishop Auckland
Bupa Dental Care Bishop Auckland, Market Place, Bishop Auckland
Bupa Dental Care Durham, Gilesgate, Durham
Bupa Dental Care Pelton, Wheldon Terrace, Pelton
Bupa Dental Care Shildon, Lime Tree House, St John's Road, Shildon
Burnopfield Dental Practice, Busty Bank, Burnopfield
(completed) Castle Dene Surgery, Peterlee
(completed) Castle Dental Practice Limited, Galgate, Barnard Castle
Castle View Surgery Limited, North Road, Durham
Cestria Dental Practice, High Chare, Chester Le Street
Chilton Dental Health Centre, Norman Terrace, Chilton
(completed) Claypath Dental Practice, Claypath, Durham
The Cosmetic Dental Clinic (Durham), 7 Old Elvet, Durham
Coundon Dental Health Centre, Victoria Lane, Coundon
Coxhoe Dental Practice, Blackgate East, Coxhoe
Dental Avenue, Clyde Terrace, Spennymoor
Dental Practice, 177 Medomsley Road, Consett
Derwent Street Dental Practice, Derwent Street, Consett
Dunelm Orthodontics, The Cross Gate Centre, Durham

Durham Dental Implant Suite, Meadowfield Industrial Estate, Durham
Elvet Dental Practice, 18 Old Elvet, Durham
Esh Winning Dental Health Centre, Station Avenue, Esh Winning
(completed) Ferryhill Dental Health Centre, Old Sorting Office, Durham Road, Ferryhill
(completed) Framwellgate Dental Surgery, Framwellgatemoor
Honour Health Stanley, Front Street, Stanley
Kingsgate Dental practice, Church Street, Durham
Murton Dental Practice, Woods Terrace East, Murton
Mydentist Consett, Front Street, Consett
Mydentist Durham, Framwellgate Bridge, Durham
Mydentist Peterlee, Yoden Way, Peterlee
Mydentist Stanley, Clifford Road, Stanley
Myorthodontist Durham, Hawthorn Terrace, Durham
Northern Smiles, Mill Street, Crook
Number One Dental Surgery, Durham Road, Consett
Pelton Lane End Dental Practice, Pelton Lane Ends, Pelton
Princess Road Dental Practice, Seaham
Queensway Oral Surgery-Ferryhill, Queensway, Ferryhill
Queensway Orthodontics North Limited, Middle Street, Consett
Queensway Orthodontics East Limited, Newgate Street, Bishop Auckland
Seaham Smiles, Maureen Terrace, Seaham
Sedgefield Dental Health Centre, North End, Sedgefield
Sedgefield Dental Practice, Front Street, Sedgefield
Shildon Smile Care, Main Street, Shildon
(completed) Shotley Bridge Dental Care, Blackhill
Smile in Durham, New Durham Road, Annfield Plain
Spennymoor Dental Health Centre, Whitworth Terrace, Spennymoor
Stanhope Dental Health Centre, Dales Street, Stanhope
Stoney Field Dental Practice, Station Road, Stanley
Trimdon Dental Health Centre, Wynyard Road, Trimdon Station
(completed) Wentworth House Dental Practice, Seaside Lane, Easington Colliery
West Auckland Dental Practice, Front Street, West Auckland
Westlands Dental Studio, Front Street, Lanchester
(completed) Willington Dental Centre, High Street, Willington
(completed) Woodham Dental Surgery, Newton Aycliffe
Wray and McKenna Limited, The Avenue, Wheatley Hill

1.c - Please select your practice

Advanced Care 2.0 Ltd, 29-30 Dean Terrace, Ryton
Bensham Dental Practice, 24 Saltwell Road, Gateshead
Dr Mohammed Bholah - Spoor Street, 1 Spoor Street, Dunston
Blaydon Dental Practice, Dunsopp House, Lucy Street, Blaydon on Tyne
Bupa Dental Care Gateshead, 443 Lobley Hill Road, Gateshead
Carew Dental Limited, Houghton House, Team Valley Trading Estate
Chapman Dental Solutions, 84 Front Street, Whickham
Crawcrook Dental Practice, 5 Beech Grove Terrace, Ryton
Crescent Dental Care, 3-4 The Crescent, Dunston
(completed) Deckham Dental Practice, 294 Old Durham Road
(completed) Deneholme Dental Practice, Birtley
Denmark Street Dental Practice Limited, 2 Denmark Street, Gateshead
Durham Family Dental Practice, 566a Durham Road, Gateshead
Durham Family Dental, 677 Durham Road, Low Fell
Gateshead Dental Care, 27a Jackson Street, Gateshead
Greencroft Lodge Dental Practice, Strathmore Road, Rowlands Gill
(completed) Indental Practice, Fewster Square, Felling
Indental Orthodontics, 25 Fewster Square, Gateshead
(completed) Low Fell Caring Dental Practice, The Lodge
Mydentist - Durham Road - Low Fell, 323 Durham Road, Low Fell
Mydentist - Neale Terrace - Birtley, 4 Neale Terrace, Birtley
Mr Pravinkumar P Nana - Winlaton, 16a The Garth, Front Street, Winlaton
New Biggin Villa Family Dental Clinic, 265 Kells Lane, Gateshead
(completed) Park View Family Dental, Felling
Pelaw Dental Clinic, 1 Musgrave Terrace, Gateshead
(completed) Perfect Smile Blaydon, Dunsopp House, Blaydon

Perfect Smile Gateshead, 15 Regent Terrace, Gateshead
Ryton Dental Clinic, 29 Dean Terrace, Ryton
Sunniside Dental Practice, 10 Sun Street, Sunniside
Valley Shopping Village Dental Practice, Kingsway, Team Valley
Village Dental Practice, 44a Front Street, Whickham
(completed) Whickham Dental Practice, 26 Front Street
The Wrekenton Dental Practice, 13 Longbank, Wrekenton

1.d - Please select your practice

(completed) Bentley Mathieson Dentalcare, Stamford House
DentalCare Seaton Carew, 48b Elizabeth Way, Seaton Carew
(completed) Elliott McCarthy Dental Care, Hartlepool
Grace Dental Care Partnership, 60 Avenue Road, Hartlepool
Hartlepool Dental and Implant Centre - Northgate, 29 Northgate, Hartlepool
Hartlepool Dental and Implant Centre - Owton Manor Lane, 212 Owton Manor Lane, Hartlepool
Hartlepool Dental and Implant Centre - Victoria Road, 34 Victoria Road, Hartlepool
Health and Smile Dental Practice, Easington Road, Hartlepool
Mydentist - Grange Road - Hartlepool, 4 Grange Road, Hartlepool
Select Orthodontics, 48b Elizabeth Way, Seaton Carew
Smith and Associates, 223 Stockton Road, Hartlepool

1.e - Please select your practice

(completed) Berwick Hills Dental Practice, Middlesbrough
(completed) Bupa Dental Care Coulby Newham, Parkway Centre, Coulby Newham
Cleveland Orthodontics, 32-36, Baker Street, Middlesbrough
(completed) D C Daniels - Linthorpe, 61 Roman Road
D C Daniels - North Ormesby, 8 Trinity Mews, North Ormesby
Genix Healthcare Middlesbrough Limited, 222 Linthorpe Road, Middlesbrough
Hemlington Dental Surgery, 18 The Viewley Centre, Hemlington
(completed) The Independent Dental Practice, 282 Acklam Road
M J C Newbould, 312 Linthorpe Road, Middlesbrough
(completed) Marton Dental Practice, Stokesley Road
Mydentist - Marton Road, 153 Marton Road, Middlesbrough
Mydentist - Martonside Way, Yttrim House, Martonside Way, Middlesbrough
Mydentist Ormesby, 4 Cargo Fleet Lane, Ormesby
(completed) Select Dental Care, 127 Borough Road
Teeside University, Borough Road, Middlesbrough
Westmount Dental - Middlesbrough, 131-133 Acklam Road, Middlesbrough

1.f - Please select your practice

(completed) Acorn Dental Surgery, 67 High Street, Gosforth
Acorn Dental Surgery, 44 Acorn Road, Jesmond
Aesdent, 6 Brandling Park
Angel Dental Care - Newcastle, 6 Frenton Close, Chapel House
Atkinson Dental Practice, 129 Osborne Road
Brunswick Dental Practice, Darrell Street, Brunswick Village
Bupa Dental Care Kelvin Lodge, 77 Elmfield Road
City Dental Newcastle, Coach House, 39 Coach Lane, Hazlerigg
D.P. Rundle Dentalcare, 18 Netherby Drive, Fenham
D.P. Rundle Dentalcare, 522 Denton Road, Denton Burn
Dental Hospital, Richardson Road
Dental Practice 2, 78 Kenton Lane, Gosforth
Dental Solutions Gosforth, 22 Lansdowne Terrace, Gosforth
(completed) Dr Alec Waugh and Associates Dental Surgeons, 13 Jesmond Vale Terrace
Fawdon Dental Practice, 1 Station Cottages, Fawdon
Gosforth Dental Surgery, 85-87 High Street, Gosforth
Gosforth Smiles Clinic, 55a Church Road, Gosforth
Grainger Park Dental Practice, 3 Grainger Park Road
Great Park Dentistry, Unit 3, Middleton North, Wagonway Drive
Heaton Dental Clinic, 392 Chillingham Road, Heaton
Honour Health Jesmond, 90 Osborne Road
Kingston Park Advanced Dentistry, 1 Stuart Court
Kingswalk Dental Practice - Leazes Park Road, 50 Leazes Park Road

M I Bholah Dental Practice, 64 Salters Road, Gosforth
Mr Robert Wain - Dovetail, Amy House, 61 Blandford Square
Mydentist - Heaton Road - Newcastle Upon Tyne, 37 Heaton Road
Mydentist - Heaton Smile - Newcastle- Upon- Tyne, 34 Heaton Road
Mydentist - Springfield Road - Newcastle, Springfield Road
Mydentist Advanced Oral Health Centre - Kenton Road, 1-2 Kenton Park Shopping Centre
Mydentist-Welbeck Road-Walker, 572 Welbeck Road
Neo Orthodontics Quayside, 98-100, The Close
Neo Orthodontics West Road, Unit 3, Condercum House, 171 West Road
(completed) Newburn Dental Surgery, 1-3 High Street
Newcastle Dental Care, 11 Ridley Place
(completed) No1 Victoria Terrace Dental Clinic, 1 Victoria Terrace
Pure Orthodontics, 48a, Osborne Road
Queensway Dental Clinic-Jesmond, 13 Eslington Terrace
Reid Family Dental Practice, 11 Salters Road, Gosforth
Saville Dental Practice, Floor 1, 6 Saville Place
Shakespeare Street Dental Practice, Adelphi Chambers, 20 Shakespeare Street
Simpson and Nisbet Dental Centre, 43 Osborne Road
Smmmile, 95-97, Grainger Street
South View dental centre, 46 South View, West Denton
(completed) St Mary's Dental Practice, 3 St Marys Place
The Cosmetic Dental Clinic, 2 Eldon Square
The Dental Care Clinic, 369 Stamforham Road, Westerhope
(completed) The Dental Practice, 137 Kenton Road, Gosforth
(completed) The Grange Dental Centre, 59-61 Great North Road, Gosforth
The Victoria Buildings, Heaton Park View, Heaton
Tyneview Dental Practice, 1 Whickham View, Benwell
Watch Dental Gosforth, 2 Redesdale Avenue, Gosforth
Westgate Dental Practice, 7 Graingervile North, Fenham
Windmill Heaton Orthodontics, 37A Heaton Road

1.g - Please select your practice

(completed) Alistair Bartlett Dental Practices - Hadrian Park
(completed) Alma Dental Practice, 3 Alma Place, North Shields
Bupa Dental Care North Shields, 102 Bedford Street, North Shields
CFE Dental, 242 The Broadway, North Shields
Denbigh Dental Practice, 2 Denbigh Avenue, High Howdon
Dentists@146, 146 Whitley Road, Whitley Bay
Dr Sally Ann Jones, 97 Park Road, Wallsend
Ewan Bramley Dental Care, The Richard Irvin Building, Union Road, North Shields
Genix Healthcare Whitley Bay Ltd, 275 Whitley Road, Whitley Bay
(completed) Great North Dental Care, Wideopen (completed)
Horizon Dental Clinic, 2a Kenilworth Road, Monkseaton
(completed) Laburnum Dental Practice, Wallsend (completed)
Marine Avenue Dental Practice, 44 Marine Avenue, Whitley Bay
Melita Dental Practice Limited, 18 Ilfracombe Gardens, Whitley Bay
Monkseaton Dental Practice, 6 Crawford Place, Monkseaton
mydentist Killingworth, Unit 20-21 The Killingworth Centre
Mydentist Wallsend, 16-18 Laburnum Avenue, Wallsend
New Smile Company, 109A Heaton Terrace, North Shields
Osborne Family Dentists - North Shields
Osborne Orthodontics, 3 Nile Street, North Shields
Rosebrough Dental Practice, Four Lane Ends
(completed) Shiremoor Dental Practice, 1 Lesbury Avenue
(completed) The Dental Practice @ No. 24, North Shields
The Family Dental Practice, 31 Tynemouth Road, Wallsend
(completed) The Villa Dental Practice, Regents Terrace, Billy Mill
The Whitley Dental Clinic, 48 Roxburgh Terrace, Whitley Bay
(completed) Verne Road Dental Practice, North Shields
Watch Dental Clinic - Benton, 2 Manor Road, Benton
Windmill North Tyneside Orthodontics, Unit 4, Rake House Farm, Rake Lane

1.h - Please select your practice

23 Cosmetic Dental Clinic, 25 Oldgate, Morpeth
Abbey Dental Practice, 9 Beaumont Street, Hexham
(completed) Alistair Bartlett Dental Practices - Ashington
Alnwick Dental Practice, 3 Bondgate Without, Alnwick
(completed) Amble Dental Practice, 45 High Street
Battle Hill Dental Practice, 11 Battle Hill, Hexham
Bedlingtonshire Dental Practice, 30 Front Street East, Bedlington
Beech Cottage Surgery, 38 Hill Street, Corbridge
Berwick Smile Dental Care, 57-63 West End, Tweedmouth
(completed) Blyth Family Dental Practice, 42 Marine Terrace
(completed) Brucegate Dental Practice, Berwick Upon Tweed
Bupa Dental Care Darras Hall, Broadway, Ponteland
Bupa Dental Care Morpeth, 23 Newgate Street, Morpeth
Burnett Dental Group, 8 Church Street, Cramlington
Butterworth Dentist Limited, 17 Bondgate Without, Alnwick
Corbridge Dental Practice, 32 Princes Street, Corbridge
Cramlington Dental Centre, Quarryside House, Middle Farm Square, Cramlington
Dental Surgery, 17 Bell Villas, Ponteland
Family Dental Practice, 44 Front Street, Prudhoe
Genix Healthcare Alnwick Ltd, Unit 10, Cawledge Business Park, Hawfinch Drive, Alnwick
Hencotes Dental Practice, 7 Hencotes, Hexham
Hexham Dental Clinic, 17 Battle Hill, Hexham
(completed) Highgate House Dental Practice, Bedlington
Honour Health Ponteland, 31 Bell Villas, Ponteland
Horizon Dental Clinic Blyth, 58 Beaconsfield Street, Blyth
Jason Old dental practice, The Gables Health Centre, 26 St Johns Road, Bedlington
Jason Old dental practice, Widdrington clinic, Grange Road, Widdrington, Morpeth
(completed) JRP Jones & Associates Limited, 4A Bridge Street, Morpeth
Linden Cottage Dental Practice, High Market, Ashington
Louise Hunter & Associates, 34 West Road, Prudhoe
Market Street Dental Practice, 15 Market Street, Alnwick
Mydentist - Cowpen Road - Blyth, 323 Cowpen Road, Blyth
MyDentist - Waterloo Road - Blyth, 72a, Waterloo Road, Blyth
Mydentist - Woodhorn Road - Ashington, 32 Woodhorn Road, Ashington
Neo Orthodontics Alnwick, Unit 2 Towergate, Lagny Street, Alnwick
Neo Orthodontics Ashington, 79 Station Road, Ashington
Newbiggin Dental Practice, 1 Gibson Street, Newbiggin By The Sea
Northern Smiles Haltwhistle, 5 Station Court, Haltwhistle
Princes Street Dental Practice Limited, Hedley House, Princes Street, Corbridge
Prudhoe Dental Practice, 78 Front Street, Prudhoe
(completed) Rivenhall Dental Practice, Cramlington
Rothbury Dental Practice, Whitton Bank Road, Rothbury, Morpeth
The Dental Room Belford, 54B High Street, Belford
The Village Dentists Limited, The Old Church, Village Road, Cramlington
(completed) Tweedmouth Dental Clinic, Shielfield Terrace, Tweedmouth

1.i - Please select your practice

Banksdental, 106 High Street, Marske-by-the-Sea, Redcar
Church Street Dental Practice, 85 Church Street, Guisborough
Coatham Dental Practice, 28 Coatham Road, Redcar
Dental Care Centre, 32 Queen Street, Redcar
Dental Surgery, Arbroath House, High Street, Loftus
(completed) Eston Dental Practice, 15 Jubilee Road, Middlesbrough
(completed) Family Dental Practice, Unit 2, Roseberry Shopping Centre, Roseberry Road, Redcar
Genix Healthcare Marske Ltd, 16 The Wynd, Marske-by-the-Sea, Redcar
Loftus Dental Practice, 27 West Road, Loftus, Saltburn By The Sea
(completed) The Mews Dental Practice, Guisborough
mydentist - Cleveland Retail Park, Cleveland Retail Park, Skippers Lane Industrial Estate
mydentist - Highcliffe View - Westgate, 1 Highcliffe View, Westgate, Guisborough
(completed) Normanby Dental, 13-17 High Street
(completed) Park Avenue Dental Practice, Redcar
Mr David Power - Marske-by-the-Sea, 106 High Street, Marske-by-the-Sea, Redcar
Redcar Family Dental Centre, 54 Lord Street, Redcar
Roseberry Dental Practice, 118 Westgate, Guisborough

Saltburn Dental Practice, 11 Dundas Street East, Saltburn By The Sea
(completed) Skelton Dental Practice, 171 High Street, Skelton
Vitality Dental Practice Guisborough, Cleveland Gate Business park, Rectory Lane, Guisborough

1.j - Please select your practice

(completed) 1A Dental Practice Partnership, 74 Fowler Street, South Shields
(completed) Boldon Lane Dental Practice, 51-53 Boldon Lane, South Shields
(completed) Cleadon Village Dental Practice, 30 Front Street, Cleadon
Dencall - Palmers Community Hospital, The Palmer Community Hospital, Wear Street, Jarrow
(completed) Dental Surgery, Marsden Road Health Centre
(completed) East Boldon Dental Practice, 6 St Bedes
(completed) Flagg Court Dental Practice, Flagg Court Primary Care Centre
(completed) Grange Road West Dental Practice, Jarrow
(completed) Hebburn Dental Clinic, 4 Park Road, Hebburn
Mr Devanand Isukapatla, 147 Prince Edward Road, South Shields
Mydentist - advanced oral health & orthodontic centre, 78 Dean Road, South Shields
Mydentist - Albert Road - Jarrow, 299 Albert Road, Jarrow
Mydentist - Marina Terrace - Whitburn, 1 Marina Terrace, Whitburn
Mydentist - Station Road - Hebburn, 15-17 Station Road, Hebburn
(completed) Mydentist - Sunderland Road - South Shields
Mydentist - Westoe Road - South Shields, 16-22 Westoe Road, South Shields
NDC South Shields, 2 Belle Vue Crescent, South Shields
North Road Dental Surgery, 22 North Road, Boldon Colliery
(completed) Perfect Smile Hebburn, 88 Victoria Road West
South Tyneside Orthodontic Centre Ltd, 8 Thomas Street, South Shields
(completed) St Michael's Dental Practice, 158 Westoe Road, South Shields
Westmount Dental Surgery @ Jarrow, 22 Bede Burn Road, Jarrow

1.k - Please select your practice

(completed) Alpha Dental Studio Fairfield
Belasis Dental Practice, Unit 19d Manor Way, Belasis Hall Technology Park, Billingham
Bishopton Lane, 21 Bishopton Lane
Bupa Dental Care Stockton, 5-7 Yarm Road
Bupa Dental Care Yarm, 4 Healaugh Park, Yarm
(completed) Burgess & Hyder Billingham, Low Grange Community Centre, East Avenue, Billingham
Enhance Dental Care, 111 High Street, Yarm
Grace Dental Care -Windlestone Road, 19a, Windlestone Road, Billingham
Grace Dental Care, 49 Tunstall Avenue, Billingham
Grace Dental Care, North Tees Hospital, Hardwick Road
Grange Dental Practice, 384-386, Norton Road
Grange Dental Practice, 431 Norton Road
(completed) Hardwick Dental Practice, 50 Hardwick Road
Identity Dental Care, 78 Wolviston Road, Billingham
Lanehouse Road Dental Surgery, 77 Lanehouse Road, Thornaby
Leven Vale Dental Practice, Low Lane, High Leven, Yarm
McCormick & Harrington Limited, 69-71 Queensway, Billingham
(completed) Mydentist - Greenside - Barwick
(completed) Mydentist - Lysander House - Stockton on Tees
(completed) MyDentist - Rimswell Parade - Stockton On Tees
Mydentist - Station Road - Eaglescliffe, 13 Station Road, Eaglescliffe
Myton Park Dental Centre, Myton Road, Ingleby Barwick
Norton Village Dental, 111 High Street, Norton
Queensway Dental Clinic-Billingham, 170 Queensway, Billingham
Queensway Orthodontics Yarm, 17a High Street, Yarm
Queensway Orthodontics, 170, Queensway, Billingham
Roseworth Dental Centre, 73 Ragpath Lane
Smile Spa Limited, 5 Innovation Court, Yarm Road
Tennant Street Dental Practice, 3-4 Tennant Street
The Dental Healthcare Centre And Cleveland Cosmetic And Dental Implant Clinic, 21 Wellburn Road, Fairfield
The Poplars Dental Practice, 104 High Street, Yarm
(completed) Thornaby Dental Centre, 31 Allensway
Wynyard Dental, 134 The Stables, Wynyard, Billingham
Wynyard Dentistry, Unit 1, Wynyard Business Village, Wynyard, Billingham

Yarm Lane Dental Surgery, 59 Yarm Lane

1.l - Please select your practice

Breeze Dental @ Ryhope, 1 Burdon Lane
Breeze Dental Chester Road, 180 Chester Road
Browns Dental Surgery, Health Centre, The Galleries, Washington Centre, Washington
Bupa Dental Care Sunderland, 495 Hylton Road, Pennywell
Bupa Dental Care Wessington, Unit 1 B, Hylton Park
Chester Road Dental Practice, 217 Chester Road
Churchview Dental Practice, 54 High Street, Easington Lane, Houghton Le Spring
City Dental, 57 Dovedale Road, Seaburn Dene
Dairy Lane Dental Practice, Dairy Lane, Houghton Le Spring
Dencall - Sunderland Royal Hospital, Kayll Road
Dental Surgery, 2 Grange Terrace, Stockton Road
Eden Villas Dental Practice, 6 Eden Villas, Washington
(completed) Fence Houses Dental Practice, 68 Station Avenue North
Frederick Street Family Dental Practice, 55 Frederick Street
Fulwell Clinic, 2 Atkinson Road, Fulwell
Fulwell Dental Practice, Fulwell Dental Pracice, 162 Fulwell Road
Glendale House, 10 Church Street, Houghton Le Spring
Grangetown Family Dental Health Centre, 41 Windsor Terrace
Hetton Dental Practice, 84 Station Road, Hetton Le Hole, Houghton Le Spring
Hylton Castle Dental Care, 21 Chiswick Square, Hylton Castle
John Street Dental Practice, 50 John Street Sunderland
Mydentist - Newcastle Road - Sunderland, 154 Newcastle Road
Mydentist - Orthodontic Centre - Sunderland, 26-27 Laura Street
Mydentist - The Green - Southwick, 44d The Green, Southwick
Mydentist Advanced Oral Health Centre - East Herrington, 27 Durham Road, East Herrington
Park Lane Dental Group, 11 Grange Terrace, Sunderland
Riveredge Dentistry Limited, Unit 2c, Hylton Park
(completed) The Dental Centre - Washington, 14 Front Street, Washington
Truly Dental, 35 Merle Terrace
Wearside Orthodontic Centre Ltd, 49 Frederick Street
Westmount Dental Surgery, 1 West Mount, Chester Road
