# Supplementary material for: Valuing and retaining the dental workforce: a mixed-methods exploration of workforce sustainability in the North East of England
Source: BMC Health Serv Res. 2025 May 10;25:672. doi: 10.1186/s12913-025-12803-9 (PMC12065166; doi:10.1186/s12913-025-12803-9)
Supplement: Supplementary file 3 — Supplementary Material 3. [file 12913_2025_12803_MOESM3_ESM.docx]

# Supplementary File 3: Dental Workforce Workshop Outline

# Materials

A4/A3 sheets with full diagram to act as prompts on the tables.

Flipchart/A3 sheets on walls:

Aim (pre-printed A3)

Drivers (for Part 1)

Changes (for Part 2 – add drivers from Part 1 to top of sheets)

Post-It notes.

# Introduction

In this session we are addressing the problem of recruitment and retention to the dental workforce in the North East, and how to fulfil the aim of **improving recruitment and retention**.

We’re drawing of the approach of ‘Driver Diagrams’ which some of you may have come across before, but in a simplified form.

The session will be in two halves:

In the first half we will be considering the **drivers** which are essential to achieving that aim. What are the things that can be changed in order to solve the workforce problem. Some of those things may be local, some national, or personal. We’ll be asking you to work in small groups for the first 10 minutes, and then discuss as a larger group.

In the second half, we’ll be looking to identify **changes** which can act upon those drivers. Changes may have a direct or indirect affect. We’ll look at the drivers identified in turn, and look for potential changes and how they may affect our aim.

We will be recording the whole group discussions for later transcription and analysis, so please speak one at a time.

# Part 1 – identifying drivers

1. (10 mins) This is a driver diagram – we’ve populated the aim, and we’d like you to consider the drivers. These are the **things which may be important for enabling or inhibiting workforce sustainability in the North East**. Some may be direct – primary drivers – and others indirect, but we’ll not worry too much about that distinction.

In groups of 3-4, take 10 minutes to think about potential drivers, and note down on Post-Its what these are. Probably 3 or 4 each will be plenty, but do add more if you want.

Don’t worry about distinguishing between primary and secondary drivers.

We’ll collect the Post-Its and put them on the flipcharts. You may also want to make notes on the sheets in your groups.

**[Researcher to collect Post-Its and sort into groups on flipchart paper – making note of the groups/type of driver.]**

1. (20 mins) We’ll now start recording the discussion. Looking at the first set of drivers, who would like to expand on this?

- Why it is a driver?
- How does it have a positive/negative effect?]
- Is there anything else we haven’t got yet?
- **[Researcher to revise driver labels if appropriate following discussion]**

# Part 2 – identifying changes (30 mins)

**[Researcher to add driver labels to top of new A3 sheets – one driver per sheet]**

We’re now going to walk through each of these drivers and think about ideas for change that could positively address the driver and fulfil our aim.

Again, these may be at different levels from national policy through local training to individual choices.

Write any changes on Post-Its as we’re talking and we’ll add that to these sheets. If you can indicate if it’s something for the immediate or short term, or longer term.

If you think of other drivers, do say so and we’ll make a note of that.

**[Talk through each driver in turn – if more than 3 drivers, ask participants to prioritise – probably only time for 10 mins per driver. Researcher to act as scribe, as receive any Post-Its]**

Main prompts:

What change would improve this driver?

How would that work?

Whose responsibility is that/who would pay for it?

What can be done now? / What needs to be longer term?

Are there any risks?
