# Supplementary material for: Valuing and retaining the dental workforce: a mixed-methods exploration of workforce sustainability in the North East of England
Source: BMC Health Serv Res. 2025 May 10;25:672. doi: 10.1186/s12913-025-12803-9 (PMC12065166; doi:10.1186/s12913-025-12803-9)

Supplementary File 4: Driver Diagram Template

Template driver diagram workshop provided to participants:


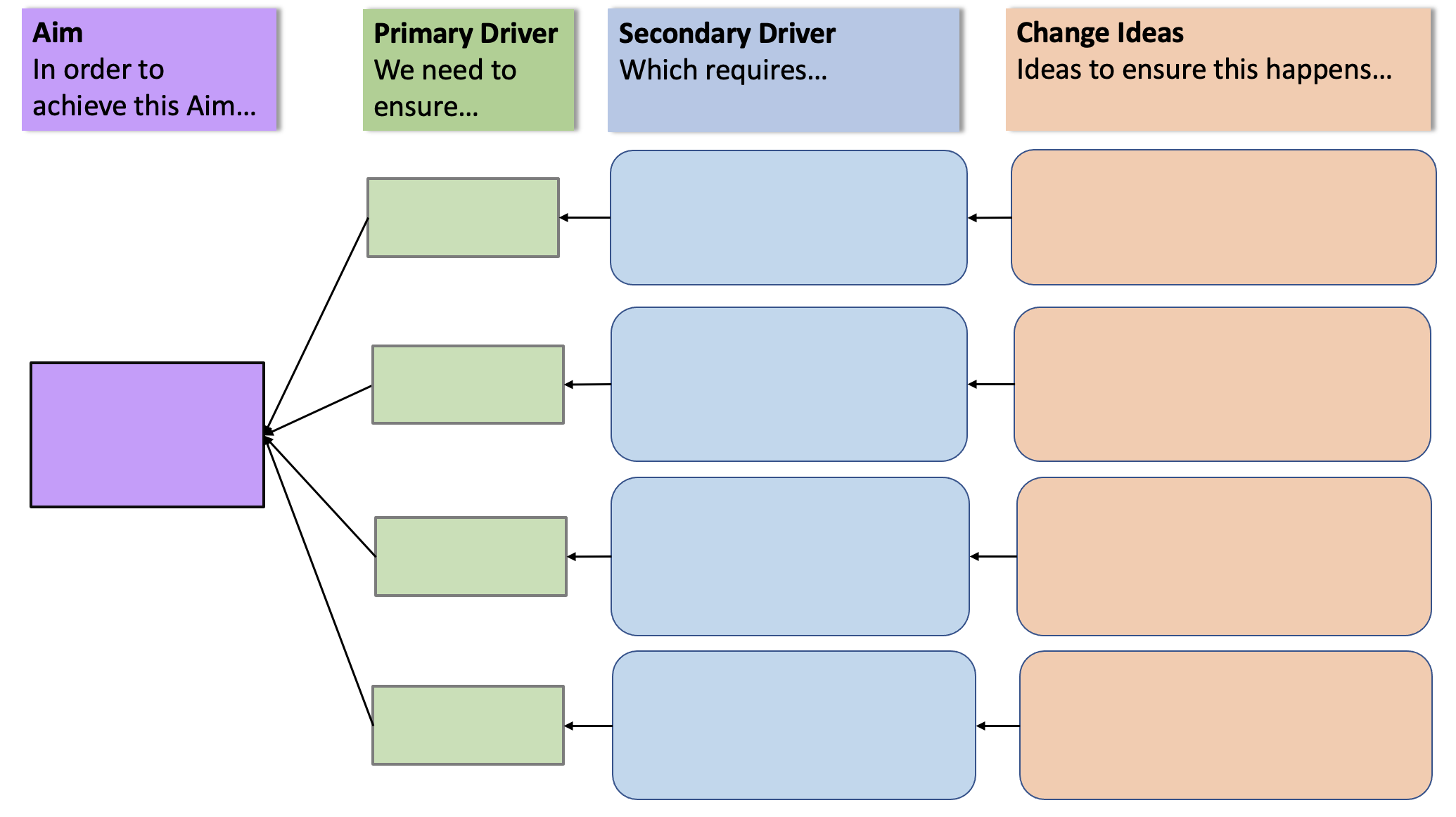

Supplement: Supplementary file 4 — Supplementary Material 4. [file 12913_2025_12803_MOESM4_ESM.docx]
