# Supplementary material for: Valuing and retaining the dental workforce: a mixed-methods exploration of workforce sustainability in the North East of England
Source: BMC Health Serv Res. 2025 May 10;25:672. doi: 10.1186/s12913-025-12803-9 (PMC12065166; doi:10.1186/s12913-025-12803-9)
Supplement: Supplementary file 5 — Supplementary Material 5. [file 12913_2025_12803_MOESM5_ESM.docx]

Supplementary File 5: Codebook

| Code NO | Code | Description |
| --- | --- | --- |
| 1 | Career progression and opportunities | - Dentists' career development and progression opportunities - Includes decisions about CPD, upskilling and taking PG qualifications. - Dentists only – DCP progression in DCP code. |
| 2 | Work environment: NHS vs Private | - Similarities and differences between NHS and private dental practice. - Includes pay, recruitment, workload, additional administrative burden, bureaucracy |
| 3 | Professional networks, support, and collaboration | - Includes personal, professional and social support, and mentorship within professional networks. - Includes references to barriers to collaboration both within and between practices. - References interactions with commissioner and regulators. - Includes expanding local networks through multi-disciplinary and muti-agency working as well as multi-site working. |
| 4 | Financial and contract management | - Contract and financial issues, including golden handshakes and incentives for staying in NHS practice. - References to commissioning decisions and role/structure of commissioners and NHS bureaucracy. - Includes UDA value undervaluing prevention, lack of flexibility in the UDA specifically not supporting activity outside direct clinical care. - Includes financial elements of access issues and building maintenance and suitability |
| 5 | Nature of clinical work and decisions | - References to the volume and content of clinical workload in NHS practice. - Includes litigation and professional responsibility |
| 6 | Dental Care Professionals (DCPs) | - References about DCPs - progression, job satisfaction, retention, working conditions, regulation, and scope of practice (diversity/variety). - Includes improved utilisation sculpted by public demand for access and ‘feeling valued’. - Excludes pay, which is covered under the Financial and Contract Management code. - Includes references to different (more cost effective) ways of working (e.g., utilising the workforce better – using DCPs to their full scope of practice) |
| 7 | Dental Foundation Training (FDs) | - References to aspects of dental Foundation Training. - Includes availability, retention, experience/quality of the scheme, and Educational Supervisors' motivation/training. - Includes financial aspects of providing FD training, including UDA contribution by FDs and ‘tie-in’ periods. - Excludes comments on recruitment (covered by national recruitment code). |
| 8 | Undergraduate dental programmes and recruitment | - References to undergraduate dental programs. - Includes location of dental schools and transitions of newly qualified dentists. - Includes incentives to train locally and importance of integration within practices. - Includes how UG placements and engagement with UG encourage recruitment or promote FD in the area. |
| 9 | National recruitment process and marketing | - References to national recruitment processes, primarily for FD (Foundation Dentists). - Includes advertising and marketing – wider pool of recruitment. |
| 10 | Workforce Sustainability | - The importance of a stable and satisfied workforce in creating workforce sustainability - Includes reference to quality of life and work-life balance. - Includes references to recruitment specifically related to geographical considerations and staffing levels but excludes references to the national recruitment process. - Includes advantages and disadvantages of working in the North-East - Excludes remoteness as it pertains to support and collaboration covered in ‘Professional networks, support, and collaboration’ |
| 11 | Public Education and Engagement | - Includes public education and engagement via community outreach. - Includes public expectation of dentistry. - Includes access issues and the importance of oral health. |
