# Supplementary material for: Valuing and retaining the dental workforce: a mixed-methods exploration of workforce sustainability in the North East of England
Source: BMC Health Serv Res. 2025 May 10;25:672. doi: 10.1186/s12913-025-12803-9 (PMC12065166; doi:10.1186/s12913-025-12803-9)
Supplement: Supplementary file 6 — Supplementary Material 6. [file 12913_2025_12803_MOESM6_ESM.docx]

**Consolidated criteria for reporting qualitative studies (COREQ): 32-item checklist**

| **No. Item** | **Guide questions/description** | **Reported on Page #** |
| --- | --- | --- |
| **Domain 1: Research team and reﬂexivity** |  |  |
| *Personal Characteristics* |  |  |
| 1. Inter viewer/facilitator | Which author/s conducted the interview or focus group? | Focus groups were facilitated by members of the research team, specifically GV, BB, HS, PB, and MB.  PAGE 5 |
| 2. Credentials | What were the researcher’s credentials? E.g. PhD, MD | GV, PB, HS are clinical academics – GV and HS are doctors, and PB is a dentist. BB and MB are non-clinical academics, with PhDs in Psychology and Medical Education, respectively.  PAGE 6 |
| 3. Occupation | What was their occupation at the time of the study? | At the time of the study, the researchers were affiliated with Newcastle University in roles related to medical and dental education research. PB is a dental academic.  PAGE 6 |
| 4. Gender | Was the researcher male or female? | Research team contains a mix of genders – 3 women and 2 men.  PAGE 6 |
| 5. Experience and training | What experience or training did the researcher have? | The researchers have significant experience conducting qualitative research in medical and dental workforce studies.  PAGE 6 |
| *Relationship with participants* |  |  |
| 6. Relationship established | Was a relationship established prior to study commencement? | No prior relationships between researchers and participants.  PAGE 5 |
| 7. Participant knowledge of the interviewer | What did the participants know about the researcher? e.g. personal goals, reasons for doing the research | Participants were aware that the researchers were conducting a study on workforce sustainability in NHS dentistry. The aims of the study in terms of wanting to improve retention and workforce distribution were shared with participants as part of the pre-consent information.  PAGE 5 |
| 8. Interviewer characteristics | What characteristics were reported about the inter viewer/facilitator? e.g. Bias, assumptions, reasons and interests in the research topic | The researchers have a professional interest in workforce sustainability and are experienced in qualitative research methodologies.  PAGE 6 |

| **Domain 2: study design** |  |  |
| --- | --- | --- |
| *Theoretical framework* |  |  |
| 9. Methodological orientation and Theory | What methodological orientation was stated to underpin the study? e.g. grounded theory, discourse analysis, ethnography, phenomenology, content analysis | The study employed a codebook approach to thematic analysis, as described by Braun & Clarke (2021).  PAGE 5 |
| *Participant selection* |  |  |
| 10. Sampling | How were participants selected? e.g. purposive, convenience, consecutive, snowball | Convenience sampling was used to recruit dental professionals present at the events, but we did have an eye to purposive involvement across different roles (dentists, dental care professionals, and managers).  PAGE 5 |
| 11. Method of approach | How were participants approached? e.g. face-to-face, telephone, mail, email | Participants were approached at two regional events held in July 2023.  PAGE 5 |
| 12. Sample size | How many participants were in the study? | 46 participants (30 dentists, 3 dental care professionals, 13 managers).  PAGE 6 |
| 13. Non-participation | How many people refused to participate or dropped out? Reasons? | None. |
| *Setting* |  |  |
| 14. Setting of data collection | Where was the data collected? e.g. home, clinic, workplace | Data were collected at two in-person events in the North East of England (one in the north, one in the south of the region). Events were held externally – at local events spaces (hotel, and race course).  PAGE 5 |
| 15. Presence of non-participants | Was anyone else present besides the participants and researchers? | No. |
| 16. Description of sample | What are the important characteristics of the sample? e.g. demographic data, date | The sample included dentists, dental care professionals, and managers from NHS practices in the North East.  PAGE 6 |
| *Data collection* |  |  |
| 17. Interview guide | Were questions, prompts, guides provided by the authors? Was it pilot tested? | A structured discussion guide was used, incorporating a ‘driver diagram’ to focus on workforce sustainability factors. Discussion guide was reviewed by team prior to use for acceptability and understanding.  PAGE 6 AND SUPPLEMENTARY FILE 4 |
| 18. Repeat interviews | Were repeat interviews carried out? If yes, how many? | No**.** |
| 19. Audio/visual recording | Did the research use audio or visual recording to collect the data? | Discussions were audio-recorded and transcribed verbatim.  PAGE 5 |
| 20. Field notes | Were ﬁeld notes made during and/or after the inter view or focus group? | Field notes were taken by co-facilitators during the focus groups.  PAGE 5 |
| 21. Duration | What was the duration of the interviews or focus group? | Focus groups lasted between 55 and 67 minutes.  PAGE 5 |
| 22. Data saturation | Was data saturation discussed? | We do not discuss data saturation because our study aimed to capture a breadth of perspectives rather than reach theoretical saturation. Given the exploratory nature of our research and the diverse professional backgrounds of participants, our focus was on generating rich qualitative insights into workforce sustainability rather than determining when no new themes emerged. Furthermore, data saturation can be difficult to define in focus group research, where group dynamics influence the depth and range of discussions (Braun & Clarke, 2021). Instead, we ensured analytical rigor through systematic coding, team thematic development, and close analysis alongside existing literature. |
| 23. Transcripts returned | Were transcripts returned to participants for comment and/or correction? | No, transcripts were not returned to participants due to time constraints, the thematic rather than individual focus of the analysis, and the use of structured coding and cross-researcher verification to ensure accuracy. |
| **Domain 3: analysis and ﬁndings** |  |  |
| *Data analysis* |  |  |
| 24. Number of data coders | How many data coders coded the data? | Multiple researchers (GV, BB, HS, MB) collaboratively coded the data.  PAGE 5/6 |
| 25. Description of the coding tree | Did authors provide a description of the coding tree? | A coding framework was developed based on prior research and refined through iterative coding.  PAGE 5 |
| 26. Derivation of themes | Were themes identiﬁed in advance or derived from the data? | Themes were derived from the data through thematic analysis.  PAGE 6 |
| 27. Software | What software, if applicable, was used to manage the data? | Microsoft word, Microsoft Excel |
| 28. Participant checking | Did participants provide feedback on the ﬁndings? | Participants did not provide feedback on findings due to study’s focus on researcher-led thematic analysis, time constraints, and the need to maintain participant confidentiality and diversity of views across focus groups. We did receive contextual feedback on findings within the author team from practicing dentist PB. |
| *Reporting* |  |  |
| 29. Quotations presented | Were participant quotations presented to illustrate the themes/ﬁndings? Was each quotation identiﬁed? e.g. participant number | Participant quotations are presented in the results section and labelled by focus group number and participant number.  PAGES 6-10 |
| 30. Data and ﬁndings consistent | Was there consistency between the data presented and the ﬁndings? | Findings are supported by participant data and direct quotes.  PAGES 6-10 |
| 31. Clarity of major themes | Were major themes clearly presented in the ﬁndings? | Four major themes (Careers, Collaboration, Costs, and Contentment) are clearly outlined in the findings.  PAGE 6 ONWARDS |
| 32. Clarity of minor themes | Is there a description of diverse cases or discussion of minor themes? | Minor sub-themes (e.g., professional development) are discussed within the major themes.  PAGES 6-10 |
